# Robust Initialization of Active Shape Models for Lung Segmentation in CT Scans: A Feature-Based Atlas Approach

**DOI:** 10.1155/2014/479154

**Published:** 2014-10-21

**Authors:** Gurman Gill, Matthew Toews, Reinhard R. Beichel

**Affiliations:** ^1^Department of Electrical and Computer Engineering, The University of Iowa, Iowa City, IA 52242, USA; ^2^The Iowa Institute for Biomedical Imaging, The University of Iowa, Iowa City, IA 52242, USA; ^3^Brigham and Women's Hospital, Harvard Medical School, Boston, MA 02115, USA; ^4^Department of Internal Medicine, The University of Iowa, Iowa City, IA 52242, USA

## Abstract

Model-based segmentation methods have the advantage of incorporating a priori shape information into the segmentation process but suffer from the drawback that the model must be initialized sufficiently close to the target. We propose a novel approach for initializing an active shape model (ASM) and apply it to 3D lung segmentation in CT scans. Our method constructs an atlas consisting of a set of representative lung features and an average lung shape. The ASM pose parameters are found by transforming the average lung shape based on an affine transform computed from matching features between the new image and representative lung features. Our evaluation on a diverse set of 190 images showed an average dice coefficient of 0.746 ± 0.068 for initialization and 0.974 ± 0.017 for subsequent segmentation, based on an independent reference standard. The mean absolute surface distance error was 0.948 ± 1.537 mm. The initialization as well as segmentation results showed a statistically significant improvement compared to four other approaches. The proposed initialization method can be generalized to other applications employing ASM-based segmentation.

## 1. Introduction

Lung segmentation methods are required for automated lung image analysis and to facilitate tasks like lung volume calculation, quantification of lung diseases, or nodule detection. Model-based techniques, such as active shape models (ASMs), have been employed for segmenting lungs in three-dimensional CT scans [1–4] or two-dimensional chest radiographs [5–8] because they incorporate prior knowledge of anatomical shape variation into the segmentation process. Progress has been made over the years in improving the robustness and accuracy of fitting the ASMs, as described in [9] for medical images in general and in [1, 4] for lung segmentation. However, as Ibragimov et al. [9] concluded, the major drawback of ASMs lies in its initialization; for correctly converging to the target image structure, the ASM must be initialized sufficiently close to it.

Several techniques have been proposed for initializing ASMs for lung segmentation. The most straightforward ones place the ASM manually [8] or in a semiautomatic manner by annotating the lung size and position [7]. Van Ginneken et al. [5] and Wang et al. [7] adopt a multiresolution framework [10] to iteratively get closer to the target image structure. However, their method assumes that the target is within a certain distance to the initial model. Several other methods initialize the model based on detecting certain points on or in close proximity to lung anatomy. Iakovidis et al. [6] employ heuristics based on finding salient control points on the spinal cord and rib-cage along with a selective thresholding algorithm. Sofka et al. [4] detect the carina of trachea and use a hierarchical detection network to predict pose parameters of left and right lung. Wilms et al. [3] use heuristics based on the bronchial tree, Sun et al. [1] detect the rib-cage, and Gill et al. [2] predict the location of the carina and lung apex to initialize an ASM. The drawbacks of these methods are that, firstly, they depend on the quality of salient point detection. For example, it is possible for rib-cage and bronchial tree to be incorrectly identified due to local changes (e.g., disease, imaging artifacts, etc.) and the heuristics employed may not be robust to the incorrect detections. Secondly, they may not work equally well for lung images at different respiratory states such as total lung capacity (TLC) and functional residual capacity (FRC) because the heuristics used may not account for the deformation of lungs [2].

This paper presents a fast and robust method for automatically initializing an active shape model to lung CT scans by learning a feature-based atlas Ψ comprising average lung shape **ξ** and a set of representative lung features **ρ**_**r**_. The representative features **ρ**_**r**_ can then be matched to features **ρ**_**t**_ in a new image, based on which a mapping is computed to transform the average lung shape **ξ** to the new image space. This information is then utilized to calculate ASM initialization parameters. Our method is based on correspondences of generic local features identified in the CT volume, rather than a small set of specific salient points.

For computing the feature-based atlas Ψ, we adopt a feature-based alignment (FBA) method [11], which identifies generic lung features in a data-driven fashion and aligns a set of training CT images. The original FBA method operates by identifying a similarity transform between sets of 3D scale-invariant image features. While feature correspondences can be identified, the similarity transform is insufficient for describing the geometrical variation across lung respiratory states (e.g., TLC and FRC). In this paper, we extend the FBA method to affine alignment, which allows us to capture variation in lung shapes and respiratory motion within a single atlas, and demonstrate the advantages compared to the original approach.

We have evaluated our method on 190 images consisting of normal and diseased lungs imaged at different respiratory states. Comparison of results with those provided in [1, 2] shows our initialization method significantly improves lung segmentation accuracy. In order to establish the quality of affine transform produced by our approach, we also compare against a registration-based approach that provides affine alignment based on the robust block matching (RBM) method [12]. The RBM method is a well known registration method that was one of the top performers in the EMPIRE 2010 challenge, which compared 34 registration algorithms [13].

## 2. Prior Work

The presented approach builds on two main components: a collection of local scale-invariant features for image alignment and a robust active shape model for image segmentation.

Local scale-invariant features are distinctive image patches defined by location, scale, and orientation. Scale-invariant features emerged in computer vision literature as a means of repeatably detecting image structure arising from the same underlying scene or object in different images, despite global changes in translation, scale, and orientation. Feature detection operates by identifying the location and scale of image patches maximizing a function of saliency, for instance, the magnitude of Gaussian derivatives in scale [14] or space [15]. Once identified, distinctive intensity patches are be encoded and robustly matched between images despite geometrical deformations or missing or occluded structure. Extensive comparisons have shown the gradient orientation histogram (GoH) descriptor to be among the most effective feature encodings [16], in particular rank-ordered variants [17] in terms of achieving correct correspondences. Scale-invariant feature methods have been generalized to 3D medical image context, and this work adopts the approach of Toews and Wells [11] where 3D features are detected extrema in a difference-of-Gaussian scale-space pyramid, which are encoded by a rank-ordered GoH descriptor.

For model-based lung segmentation, we utilized a combination of robust active shape model (RASM) and subsequent graph-based optimal surface finding (OSF) algorithm [1]. The RASM employs a point distribution model (PDM), which is constructed separately for left and right lungs, and the subsequent segmentation steps are carried out separately as well. Below we briefly review the steps employed for initializing the PDM of the RASM.

Given *N* training CT images {*C*_1_, *C*_2_,…, *C*_*N*_} and presegmented training lung shapes (left or right) {**S**_1_, **S**_2_,…, **S**_**N**_}, a set of *k* corresponding points or landmarks are automatically identified for each training shape: **S**_**i**_ = {(*x*_*i*,1_, *y*_*i*,1_, *z*_*i*,1_),…, (*x*_*i*,*k*_, *y*_*i*,*k*_, *z*_*i*,*k*_)}. A PDM is constructed by aligning the training shapes **S**_**i**_ using Procrustes analysis and performing principal component analysis (PCA) on them. Let the aligned shapes be denoted by **S**_**i**_^**a**^ = {(*x*_*i*,1_^*a*^, *y*_*i*,1_^*a*^, *z*_*i*,1_^*a*^),…, (*x*_*i*,*k*_^*a*^, *y*_*i*,*k*_^*a*^, *z*_*i*,*k*_^*a*^)}. A new shape **S** can be now represented in terms of the mean shape, ***μ***_pdm_ = (1/*N*)∑_*i*_ **S**_**i**_^**a**^, using the linear model:

(1) $$\begin{array}{c} S=\mu_{\text{pdm}}+Pb, \end{array}$$

where *P* denotes the shape eigenvector matrix and **b** represents the shape coefficients [1]. To start the RASM fitting process, the mean shape ***μ***_pdm_ is initialized in the target image space based on pose parameters *T* comprising of isotropic scale, location, and rotation. In this paper, we present a novel approach to compute the pose parameters *T* for RASM initialization.

## 3. Methods

In our method, we generate a feature-based atlas based on aligning the training CT images *C*_*i*_ using the extended FBA method (Section 3.3). Utilizing the alignment information, the individual training lung shapes **S**_**i**_ can be transformed to the atlas space and averaged using existing landmarks. The main purpose of building an atlas is to embed an average lung shape with a set of representative lung features that can then be matched to features in a new CT image.

Our method for automatically initializing a RASM to lung CT images primarily consists of 3 steps: (i) building an atlas Ψ = {**ξ**, **ρ**_**r**_} comprising average lung shape **ξ** and a set of representative lung features **ρ**_**r**_ (Section 3.1); (ii) transforming **ξ** to the new subject image space based on matching features between the new image and **ρ**_**r**_; followed by (iii) computing the pose parameters *T* (Section 3.2) for RASM initialization.

### 3.1. Feature-Based Atlas

**Figure 1:**
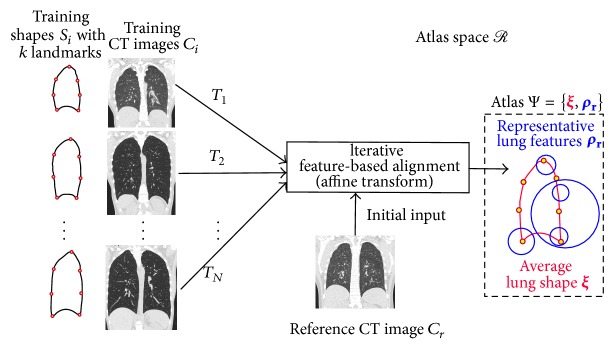
Generation of the feature-based atlas Ψ, comprising the average lung shape **ξ** and representative lung features **ρ**_**r**_ in the atlas space *R*. Note that the features **ρ**_**r**_ are derived from training CT images and encode both appearance and geometric properties [11]. For sake of clarity, the above process is shown only for the right lung.

To build an atlas Ψ, we derive an average lung shape **ξ** and associated set of representative lung features **ρ**_**r**_ as follows (Figure 1).

**Figure 2:**
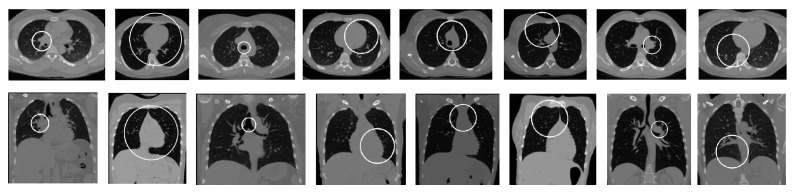
Example of representative lung features **ρ**_**r**_. Each column shows a single feature in axial and frontal view.

- 3D scale-invariant image features **f**_**i**_ are extracted from all training CT images *C*_*i*_. One of the training images is chosen as the reference image *C*_*r*_. Let the features in *C*_*r*_ be denoted by **f**_**r**_.
- The FBA method (Section 3.3) is employed to find an affine transform *T*_*i*_ from a matching set of features in **f**_**i**_ and **f**_**r**_.
- Training features **f**_**i**_ are transformed from training to atlas space *R* via *T*_*i*_. Let these be denoted by *T*_*i*_ **f**_**i**_.
- A modeling step [18] computes a new representative set of features **f**_**r**_ from the set {*T*_*i*_ **f**_**i**_}_*i*=1_^*N*^.
- Steps (b)–(d) are repeated until convergence, that is, when the Frobenius norm of the transformation matrix difference max⁡||*T*_*i*_^*t*^ − *T*_*i*_^*t*−1^|| is zero [18]. This yields a representative set of lung features **ρ**_**r**_ = **f**_**r**_ (Figure 2). Note that the iterative group-wise alignment procedure reduces the dependency on the selection of the reference image [18].
- Training shapes {**S**_**i**_}_*i*=1_^*N*^ are transformed to the atlas space by using the affine transforms *T*_*i*_. We leverage existing landmarks across shapes **S**_**i**_ and take their average to produce the average lung shape **ξ** = (1/*N*)∑_*i*=1_^*N*^ *T*_*i*_ **S**_**i**_. Note that same transformations *T*_*i*_ are used to separately compute the average for left and right lung shapes.

### 3.2. Computing Pose Parameters

**Figure 3:**
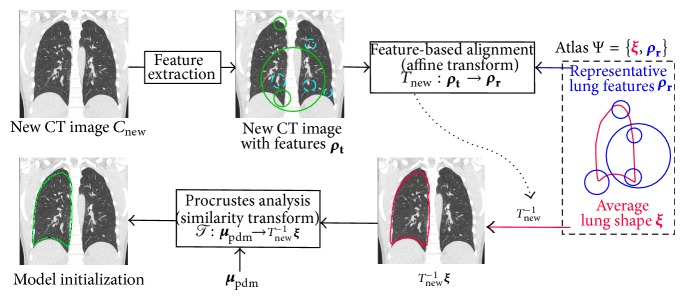
Schematic diagram showing the model initialization process for a new CT image. Features **ρ**_**t**_ are shown superimposed on the coronal slice of a CT image. Features matching with representative lung features **ρ**_**r**_ are marked with a solid line while the others are marked with a dashed line. For sake of clarity, the above process is shown only for the right lung.

3D scale-invariant image features **ρ**_**t**_ are extracted from the new CT image *C*_new_ and matched to the representative lung features **ρ**_**r**_ (Figure 3). The matching set of features output an affine transform *T*_new_ that describes how *C*_new_ can be transformed to the atlas space *R*. Since we already have a representation of the average lung shape in *R*, it is transformed to the new subject image space using *T*_new_^−1^ **ξ**. Note that the same transformation *T*_new_^−1^ is used for transforming both the left and right lung shapes.

RASM model fitting starts by placing the average PDM shape ***μ***_pdm_ (1) in the image space. The pose parameters *T* are obtained by transforming ***μ***_pdm_ to the image space by means of a Procrustes analysis *T* : ***μ***_pdm_ → *T*_new_^−1^ **ξ**.

### 3.3. Extending the Feature-Based Alignment Approach

As noted earlier, our work extends the original FBA approach [11] to compute affine alignment. First we briefly describe the key step of feature matching, followed by the new procedure for affine refinement.

Feature matching begins by computing nearest neighbors (NN) between feature descriptors in the image and atlas, using the Euclidean distance similarity measure. This procedure can be computed efficiently via fast approximate nearest neighbor methods. Each NN image-to-atlas feature match provides an estimate of the global image-to-atlas similarity transform, and a highly probable global transform along with inlier image-to-atlas matches is identified via a robust probabilistic voting formulation similar to the Hough transform [11]. Note that incorrect outlier correspondences have no effect on the transform identified, and for this reason alignment is considered robust.

Once the inlier image-to-atlas matches are identified, an affine transform *T*_*i*_ is fitted between them with a least squares approach. This allows our framework to account for a higher degree of shape variability, for example, lungs in different respiratory states, and thus produce a lung shape representative of lung variation across the population. Note that the modeling step (d) in Section 3.1 requires isotropic features (i.e., spherical as against elliptical 3D shape) [11]. For that purpose, we compute the geometric mean from the scale components of the affine transform to transform the feature scale.

The original FBA approach achieves alignment across global image rotation via rotationally invariant features, where a 3D orientation is assigned to individual features based on the structure of the local image gradient. Our lung CT data exhibit only minor orientation differences between subjects due to a similar imaging protocol, and we found that assigning a fixed feature orientation results in a higher number of image-to-image correspondences and improved alignment. This may be because many pulmonary structures exhibit rotational symmetry, for example, airways, in which case orientation is inherently ambiguous. Thus, features of fixed orientation appear to be more effective than rotationally invariant features for identifying correspondences in the case of minor intersubject orientation differences.

## 4. Evaluation

### 4.1. Image Data

We selected 190 multidetector computed tomography (MDCT) thorax scans of lungs for testing from 6 different sets *S*_normal_, *S*_asthma_, *S*_COPD_, *S*_mix_, *S*_IPF_, and *S*_tumor_ with no significant abnormalities (normals), asthma (both severe and nonsevere), chronic obstructive pulmonary disease (COPD with GOLD 1 to 4), mixture of different lung diseases, idiopathic pulmonary fibrosis (IPF), and lung cancer, respectively. The total number of scans in sets *S*_normal_, *S*_asthma_, *S*_COPD_, *S*_mix_, *S*_IPF_, and *S*_tumor_ was 20, 24, 28, 26, 62, and 30, respectively. The first four datasets contained pairs of TLC and FRC images while the last two were all TLC images. The image sizes varied from 512 × 512 × 205 to 512 × 512 × 780 voxels. The slice thickness of images ranged from 0.5 to 1.25 mm and the in-plane resolution from 0.49 × 0.49 to 0.91 × 0.91 mm.

### 4.2. Experimental Setup

The PDM was built using a separate set of 75 TLC and 75 FRC normal lung scans. The average lung shape **ξ** was constructed using 50 TLC and 50 FRC scans that were a subset of those used in building the PDM. A 3D SIFT-based feature extractor [18] was used to extract approximately 2500 features in each lung image from which high density (>0 HU) structures (e.g., bones) were automatically removed. The affine feature-based alignment system (Section 3.1) grouped these into 1000 representative lung features, forming one component of our atlas.

We followed the implementation of the RASM-OSF framework for lung segmentation presented in [1] with the exception of RASM initialization. We refer to different methods evaluated in this paper based on their method of initialization: by detecting ribs (*M*_ribs_) [1], carina and apex (*M*_capex_) [2], and average lung shape **ξ** in this paper using affine FBA (*M*_FBA^Aff^_), using similarity transform provided by original FBA (*M*_FBA^Sim^_) [18], and using affine transform obtained from RBM (*M*_RBM^Aff^_) [12] (implementation: NiftyReg, http://www.cs.ucl.ac.uk/staff/m.modat).

### 4.3. Independent Reference and Quantitative Indices

An independent reference standard was generated for all test data sets by first using a commercial lung image analysis software package Apollo (VIDA Diagnostics Inc., Coralville, IA) to automatically create lung segmentations. These were then inspected by a trained expert under the supervision of pulmonologist, and segmentation errors were manually corrected.

For performance assessment, the dice coefficient *D* was computed with respect to reference segmentations to measure the accuracy of model initialization (*D*_init_) and of the final segmentation after the RASM and OSF segmentation steps (*D*_final_). In addition, the mean absolute surface distance *d*_*a*_ was calculated.

## 5. Results

Table 1 presents the average initialization (*D*_init_) and segmentation accuracy (*D*_final_) obtained by our method on different test sets. Tables 2 and 3 compare the average initialization (*D*_init_) and segmentation accuracy (*D*_final_ and *d*_*a*_) between different methods, respectively. Based on the paired Wilcoxon rank test on *D*_init_, *D*_final_, and *d*_*a*_, our method *M*_FBA^Aff^_ shows statistically significant improvement over all other methods.  Table 3 also shows the number of cases, for each method, whose final segmentation performance is below a certain Dice value.

**Figure 4:**
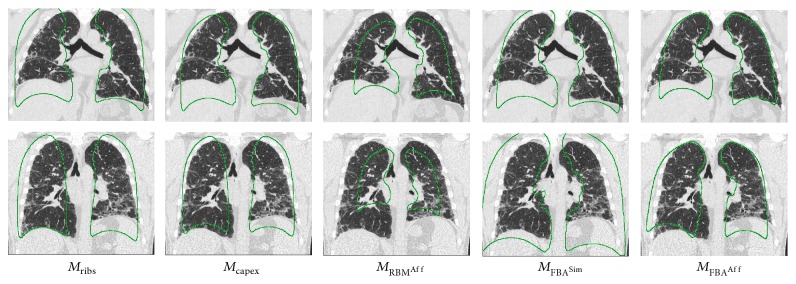
Two examples of model initializations based on different methods. The method *M*_FBA^Aff^_ shows initialization quite close to the target shape whereas the other methods are off in at least one of the examples.

**Figure 5:**
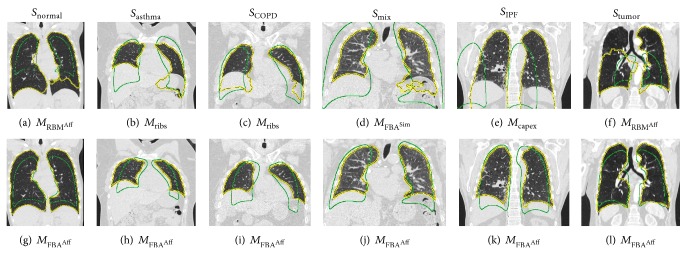
ASM initializations (green contour) and final segmentations (yellow contour) across different test sets. (a)–(f) Result of other methods for comparison with results produced with the proposed method (g)–(l).

Figure 4 shows two examples of model initialization generated using different methods. Our method *M*_FBA^Aff^_ obtains initializations very close to the target while the other methods are sometimes off. Figures 5(a)–5(f) show 6 different cases where the initialization obtained by other methods causes the segmentation to be inaccurate. In all those cases, our method (Figures 5(g)–5(l)) obtains a closer initialization and good segmentation.

## 6. Discussion and Conclusion

Compared to the four other approaches, the proposed method delivered significantly better initializations (Table 2), which also translated into significantly better overall segmentation performance (Table 3). In addition, it produces segmentations with *D*_final_ ≥ 0.8 in all cases, while all the other methods have 10 or more segmentations with *D*_final_ < 0.8. Table 1 shows that initialization performance (*D*_init_) is in a close range across test sets. Due to superior initialization, the final segmentations generated by our method converge correctly to the target structure across test sets (Figures 5(g)–5(l)).

The importance of extending FBA to affine transform is underlined by its statistically significant improvement over the version using the similarity transform (*M*_FBA^Sim^_). Moreover, it is also superior to the affine transforms produced by the RBM method (*M*_RBM^Aff^_). The reason is that RBM, like most registration methods, typically works well when lung masks are available. For example, 16 out of 20 algorithms discussed in the EMPIRE challenge paper used lung masks [13]. Generating lung masks is the goal of the presented work, and thus we did not employ any masks for the RBM method. Therefore, the affine global alignment produced by RBM can be inaccurate, resulting in low initialization accuracy (Table 2) and subsequent segmentation failures (Figures 5(a) and 5(f)).

**Figure 6:**
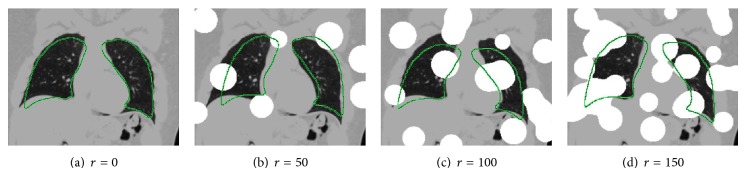
ASM initializations using *M*_FBA^Aff^_ on an image with varying degree of synthetic occlusion. The synthetic occlusion is generated by placing *r* spheres, each of radius 25 mm at random locations in the CT image. The voxels of the spheres are assigned a Hounsfield unit value of 500 HU. As can be seen, the method is robust to a large amount of occlusion.

Our method produces robust ASM initializations as demonstrated by an experiment on synthetic lung data depicted in Figure 6. Despite different levels of occlusion, the ASM is placed in close proximity to the actual lung in all cases. In addition, our method is fast and takes about 30 seconds on average, the majority of which is required for feature extraction. In comparison, *M*_RBM^Aff^_ takes 2.5 minutes, *M*_ribs_ 2 minutes, and *M*_capex_ 40 seconds on average.

We note that the mean PDM ***μ***_pdm_ could be directly transformed to the average lung shape **ξ** during the training phase. However, due to subsequent affine transformation of **ξ** (Figure 3), the isotropic scale property of the PDM would not be preserved. Future efforts will use anisotropic scale during PDM building and RASM fitting so that all calculations can be done in the affine space.

The proposed method only requires a set of training shapes with landmarks and a set of representative features learnt from training CT images to initialize an ASM in a new image. Consequently, our method can be generalized to other medical imaging applications that employ an ASM-based segmentation approach. Furthermore, it can be adapted for 2D and 4D applications.

## Figures and Tables

**Table 1:** Average initialization and final segmentation accuracy obtained by *M*_FBA^Aff^_ for each test set.

	*S*_normal_	*S*_asthma_	*S*_COPD_	*S*_mix_	*S*_IPF_	*S*_tumor_
*D*_init_	0.786 ± 0.054	0.774 ± 0.057	0.741 ± 0.059	0.717 ± 0.067	0.718 ± 0.067	0.783 ± 0.054
*D*_final_	0.985 ± 0.006	0.979 ± 0.011	0.981 ± 0.012	0.971 ± 0.017	0.963 ± 0.022	0.981 ± 0.005

**Table 2:** Comparison of median and average of initialization accuracy as well as the *P* values of a paired Wilcoxon rank test between each method and *M*_FBA^Aff^_.

	*M*_ribs_	*M*_capex_	*M*_RBM^Aff^_	*M*_FBA^Sim^_	*M*_FBA^Aff^_
Average *D*_init_	0.683 ± 0.092	0.586 ± 0.168	0.527 ± 0.125	0.707 ± 0.116	**0.746 ± 0.068**
Median *D*_init_	0.7018	0.6287	0.5236	0.7374	**0.7484**
*P* value	2.53*e*−36	4.01*e*−58	1.36*e*−63	6.97*e*−10	—

**Table 3:** Comparison of median and average of final segmentation accuracy as well as the *P* values of a paired Wilcoxon rank test between each method and *M*_FBA^Aff^_. For each method, the number of cases ^#^with Dice values below 0.9, 0.8, and 0.7 is provided.

	*M*_ribs_	*M*_capex_	*M*_RBM^Aff^_	*M*_FBA^Sim^_	*M*_FBA^Aff^_
Average *D*_final_	0.963 ± 0.056	0.944 ± 0.134	0.964 ± 0.058	0.963 ± 0.056	**0.974 ± 0.017**
Median *D*_final_	0.9784	0.9784	0.9778	0.9786	**0.9792**
*P* value	6.47*e*−05	1.18*e*−07	6.92*e*−04	6.01*e*−04	—

Average *d*_*a*_ (mm)	1.350 ± 2.456	2.271 ± 6.546	1.398 ± 3.094	1.335 ± 2.493	**0.948 ± 1.537**
Median *d*_*a*_ (mm)	0.7342	0.7441	0.7958	0.7144	**0.7108**
*P* value	7.64*e*−04	5.85*e*−07	1.24*e*−03	4.03*e*−02	—

#(*D*_final_ < 0.9)	23	31	14	23	**3**
#(*D*_final_ < 0.8)	14	23	10	11	**0**
#(*D*_final_ < 0.7)	4	15	7	4	**0**
